# Stretchable and Conductive Composite Structural Color Hydrogel Films as Bionic Electronic Skins

**DOI:** 10.1002/advs.202102156

**Published:** 2021-08-26

**Authors:** Hui Zhang, Jiahui Guo, Yu Wang, Lingyu Sun, Yuanjin Zhao

**Affiliations:** ^1^ Department of Rheumatology and Immunology Institute of Translational Medicine The Affiliated Drum Tower Hospital of Nanjing University Medical School Nanjing 210008 China; ^2^ State Key Laboratory of Bioelectronics School of Biological Science and Medical Engineering Southeast University Nanjing 210096 China; ^3^ Chemistry and Biomedicine Innovation Center Nanjing University Nanjing 210023 China

**Keywords:** bio‐inspired, electronic skin, hydrogel, PEDOT:PSS, silk fibroin, structural color

## Abstract

Electronic skins have received increasing attention in biomedical areas. Current efforts about electronic skins are focused on the development of multifunctional materials to improve their performance. Here, the authors propose a novel natural‐synthetic polymers composite structural color hydrogel film with high stretchability, flexibility, conductivity, and superior self‐reporting ability to construct ideal multiple‐signal bionic electronic skins. The composite hydrogel film is prepared by using the mixture of polyacrylamide (PAM), silk fibroin (SF), poly(3,4‐ethylenedioxythiophene):poly (4‐styrene sulfonate) (PEDOT:PSS, PP), and graphene oxide (GO) to replicate colloidal crystal templates and construct inverse opal scaffolds, followed by subsequent acid treatment. Due to these specific structures and components, the resultant film is imparted with vivid structural color and high conductivity while retaining the composite hydrogel's original stretchability and flexibility. The authors demonstrate that the composite hydrogel film has obvious color variation and electromechanical properties during the stretching and bending process, which could thus be utilized as a multi‐signal response electronic skin to realize real‐time color sensing and electrical response during human motions. These features indicate that the proposed composite structural color hydrogel film can widen the practical value of bionic electronic skins.

## Graphical abstract

A stretchable and conductive composite structural color hydrogel film with superior self‐reporting ability can be utilized as an intelligent multiple‐signal bionic electronic skin. The composite hydrogel film exhibits obvious color and electrical variation during the stretching and bending process, which makes it feasible to realize real‐time color sensing and electrical response during human motions.

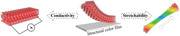

## 1 Introduction

As an essential part of artificial intelligence, electronic skins have received increasing attention and achieved remarkable progress in motion and health monitoring, disease diagnosis, implantable devices, soft robots, and so on.^[^ ^1^ ^]^ Thus, considerable research efforts are committed to developing novel materials or structures with high performance as electronic skins, aiming to realize the sensing of dynamic deformation under different physical stimuli.^[^ ^2^ ^]^ Among these materials, hydrogels are considered ideal candidates for biomimetic electronic skins due to their excellent flexibility and adjustable mechanical properties. Up to now, various hydrogels have been explored in flexible electronic devices.^[^ ^3^ ^]^ Although with many successes, most of the hydrogels are derived from synthetic polymers, and their complicated extraction or synthesis processes may cause controversial biocompatibility.^[^ ^4^ ^]^ Besides, present strategies for capturing the change of hydrogel‐derived electronic skins usually require precise and expensive instruments, which are relatively cumbersome and often limited outdoor applications that need real‐time monitoring. Therefore, new types of biocompatible polymer hydrogel electronic skin capable of self‐reporting are still anticipated.

**Figure 1:**
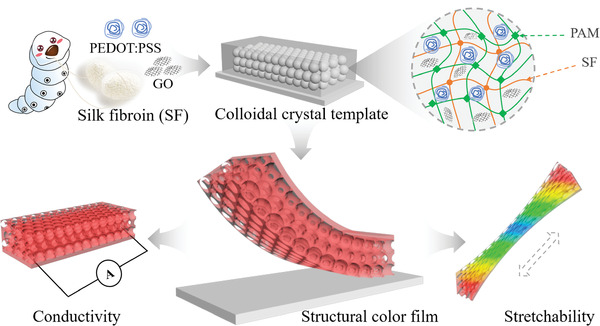
Schematic diagram. The schematic of the composite stretchable and conductive structural color hydrogel film with self‐reporting ability as electronic skins.

In this paper, we propose novel natural‐synthetic polymers composited structural color hydrogel electronic skin with the desired features, as schemed in **Figure** **1**. Silk fibroin (SF), extracted from silkworm cocoons, possess excellent biocompatibility, superior mechanical properties, low cost, and abundant sources.^[^ ^5^ ^]^ Benefitting from these advantages, silk fibroin has been widely developed in various biomedical fields such as drug delivery, tissue engineering, medical dressings, and even simple electronic skins.^[^ ^6^ ^]^ On the other hand, structural color, resulting from the interaction of light and periodically arranged nanostructures, has received extensive attention in optical devices.^[^ ^7^ ^]^ Particularly, when structural color is combined with soft hydrogels, the change of volume or shape under different stimuli can lead to visual color variation.^[^ ^8^ ^]^ This feature endows the structural color materials with prominent self‐reporting ability and exhibit crucial practical value in sensing visualization, thereby gradually attracting extensive research interest in flexible electronic skin.^[^ ^4^, ^9^ ^]^ However, the integration of silk fibroin with structural color for bionic electronic skins remains unexplored.

Herein, we fabricated a composite structural color hydrogel film with high stretchability, flexibility, conductivity, and superior self‐reporting ability to construct ideal multiple‐signal bionic electronic skins. For this purpose, polyacrylamide (PAM) and SF were mixed to negatively replicate the colloidal crystal templates, where inverse opal film with periodic nanostructures could be obtained. The resultant film was imparted with unique optical properties and vivid structural color while retaining the composite hydrogel's original stretchability and flexibility. Besides, the high conductivity of the composite hydrogel was achieved by introducing poly(3,4‐ethylenedioxythiophene):poly (4‐styrene sulfonate) (PEDOT:PSS, PP) and graphene oxide (GO) into the mixture of PAM and SF with subsequent acid treatment. It was demonstrated that the composite structural color hydrogel film had obvious color variation and electromechanical properties during the stretching and bending process. Thus, such film was utilized as a multi‐signal response electronic skin to realize real‐time color sensing and electrical response during human motions, superior to the single‐signal sensor generally reported. These results indicated that the composite structural color hydrogel film is valuable for many practical applications.

## 2 Results and Discussions

**Figure 2:**
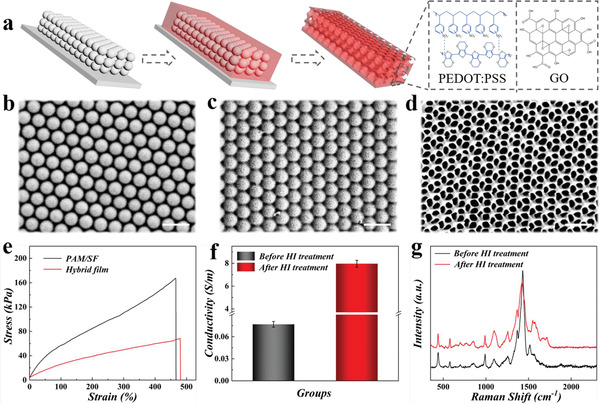
Characterizations of structural color film. a) Schematic diagram of the fabrication process of structural color film. b–d) Scanning electron microscope (SEM) images of the colloidal crystal template (b), the hydrogel hybrid film (c), and the inverse opal film (d). The scale bars are 400 nm in b, c, and d. e) The tensile properties of the PAM/SF hydrogel and composite structural color film. f) The conductivity of composite structural color film before and after HI treatment. g) Raman spectra of the composite structural color film before and after HI treatment.

In a typical experiment, the composite structural color hydrogel film was first prepared by negatively replicating the colloidal crystal templates formed during the silica nanoparticles’ self‐assembly process (**Figure** **2**a). The synthesized silica nanoparticles were dispersed in ethyl alcohol after purification, and then deposited vertically on a planar glass substrate. The highly ordered hexagonal close‐packed structures were gradually formed driven by capillary force along with the solvent volatilization. As a result, interconnected nanopores were formed between adjacent nanoparticles, which provided space for the subsequent filling of the pre‐gel solution (Figure 2b). The pre‐prepared PAM/SF/PP/GO mixed pre‐gel solution penetrated the nanopores under capillary action, followed by the polymerization under ultraviolet light to form a hybrid hydrogel film (Figure 2c). After etching the silica nanoparticles with hydrofluoric acid, a structural color film with spatially ordered porous structures was finally obtained (Figure 2d). Because the gel's thickness was higher than that of silica templates, the free‐standing structural color film showed a double‐layer form, namely the upper inverse opal structure and bottom hydrogel film (Figure S1, Supporting Information).

Due to the formation of interpenetrating networks, the addition of SF could significantly improve the mechanical properties of pure PAM hydrogel (Figure 2e). To optimize SF content, we explored the tensile property of PAM/SF composite hydrogels with different SF contents, and a volume ratio of 5:4 (PAM:SF) was found to own the superior tensile property (Figure S2, Supporting Information). Next, to impart excellent conductivity to the hydrogels, PP and GO were incorporated into the PAM/SF hydrogel successively, and the conductivity of PAM/SF hydrogels with different ratios of PP:GO was measured. It was found that when the amount of GO reached 0.1wt%, the hydrogel conductivity increased from ≈0.036 to 0.085 S m^−1^ (Figure S3a, Supporting Information). Such increase might be attributed to the separation of the PEDOT chain and the PSS chain, as well as the weakness of the two chains’ interaction caused by the functional groups on the graphene oxide sheet (such as carboxyl, hydroxyl, and epoxy groups).^[^ ^10^ ^]^ But the hydrogel conductivity decreased with the further addition of GO, potentially due to the reduction of the conductive path resulted from the excess addition of GO. Then, we immersed the composite film in a fresh hydroiodic acid (HI) solution to further improve film conductivity. On the one hand, the H^+^ in HI can effectively bind to the PPS chain to break it away from PEDOT:PSS, and the PEDOT chains would change from the coiled conformation to the uncoiled or linear conformation, facilitating the transport of inter‐chain charges; on the other hand, HI can remove the epoxy groups on GO and reduce GO to reduced‐graphene oxide (rGO) with better conductivity, as indicated in Figure S4, Supporting Information.^[^ ^11^ ^]^ Finally, the film conductivity significantly increased to 8 S m^−1^ after HI treatment, nearly 100 times higher than the untreated film (Figure 2f and Figure S3b, Supporting Information). As shown in Figure 2g, PEDOT:PSS did not show an evident Raman shift after acid treatment due to the limited effect of PSS on Raman spectra which was removed from PEDOT after HI treatment. It could be seen from the Raman spectra that most of the bands were related to the structures of PEDOT:PSS. The peaks observed at 1516, 1432, 1368, 1096, 990, 854, 701, 577, and 440 cm^−l^ all belonged to PEDOT:PSS, which also demonstrated the successful incorporation of PEDOT:PSS. Moreover, such composite hydrogel system exhibited good compressive performance that could withstand up to 60% strain without breaking, and the continuous cyclic compression test demonstrated its excellent resilience, which might be attributed to recoverable physical interactions between the components (Figure S5, Supporting Information). Besides, it was obvious that the composite hydrogel film kept its stretchable and conductive properties under different humidity conditions, as demonstrated in Figure S6, Supporting Information. Interestingly, such film could also adapt to the extreme bending angle of 180° due to its excellent flexibility, as shown in Movie S1, Supporting Information. These features provided great possibilities for such a system to move towards practical applications.

Owing to the periodically ordered inverse opal structures, the composite structural color hydrogel films possessed photonic bandgap (PBG) property, thus being endowed with bright structural colors. When incident light illuminates the material, only light with a specific wavelength could be reflected because the light propagation would be interfered by nanostructures of inverse opal films.^[^ ^12^ ^]^ Thus, the inverse opal films presented vivid structural color with the related characteristic reflection peaks. Generally, the reflection peak wavelength *λ* can be estimated by the Bragg equation:

(1) $$\lambda =1.633dn_{\mathrm{average}}$$

where *d* refers to the center‐to‐center distance between adjacent nanoparticles, and *n*_average_ is the average refractive index of the materials. According to the formula (1), the reflection peak position *λ* is only related to *d* under the condition of keeping the material composition unchanged. Therefore, it is feasible to design inverse opal films with diverse structural color based on colloidal crystal templates composed of different sizes of the silica nanoparticles. It was found that the reflection peaks of the hydrogel hybrid film and inverse opal hydrogel film both exhibited obvious redshift, which could be attributed to the increase of refractive index (Figure S7, Supporting Information). Besides, it was found that the inverse opal hydrogel film containing PP and GO was imparted with a brighter structural color compared with the pure inverse opal films, which was attributed to two dark conductive materials providing a background with stronger color contrast.

**Figure 3:**
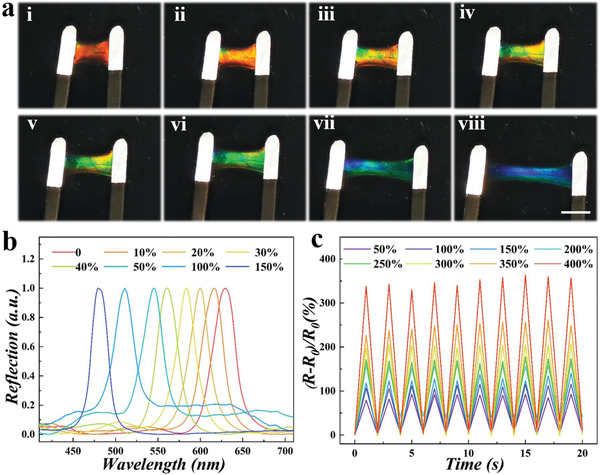
The changes of optical and electrical signals of the composite hydrogel film during the stretching process. a) Optical images of the composite hydrogel film under different strains from 0 (i), 10% (ii), 20% (iii), 30% (iv), 40% (v), 50% (vi), 100% (vii), and 150% (viii). The scale bar is 1 cm. b) The reflection peaks of composite hydrogel films with different strains. c) Relative resistance changes of composite hydrogel film during the stretching process.

Benefitting from the structural color property and the addition of conductive materials, the designed composite hydrogel structural color films owned visually dual‐signal responses. To validate these characteristics, a strain–sensitivity experiment was first conducted, as shown in **Figure** **3**. The results showed that when the composite hydrogel film was stretched from 0% to 150%, its structural color gradually varied from red to blue‐green (Figure 3a). The corresponding reflection spectra also exhibited a significant blue shift from 653 to 507 nm due to the gradual decrease in the distance between the diffraction planes (Figure 3b). Apart from the above optical signal, the resistance of the composite hydrogel film changed significantly during stretching from 0% to 400%, where *R*_0_ is the original resistance and *R* refers to the resistance in real‐time (Figure 3c). When the strain was 100%, the film's relative resistance change reached 100%, while at the stain of 400% the change reached nearly 350%, indicating that the film had a sensitive response in conductivity to a wide deformation range. To test the robustness of prepared hydrogel film, the tensile cycle tests were carried out at a strain of 50%. It was evident that both the reflection spectra change and the relative resistance change were stable and repeatable, verifying that the hydrogel film had good sensitivity, durability and resilience during the repeated stretching process (Figure S8, Supporting Information). These results demonstrated that the composite hydrogel structural color film possessed excellent electrical‐optical dual‐signal response with visualization and self‐reporting, showing the great application potential in the field of electronic skin.

**Figure 4:**
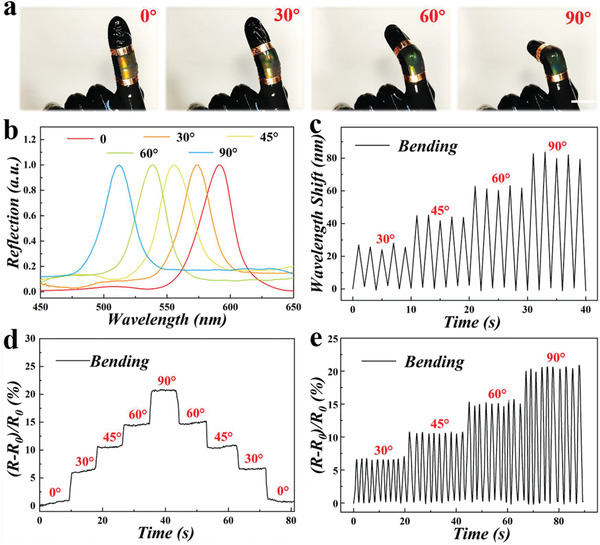
The changes of optical and electrical signals of the composite hydrogel film during the bending process. a) Photographs of the composite hydrogel structural color film with different bending angles of the finger. The scale bar is 1 cm. b) The reflection peak wavelengths of composite hydrogel films with different bending angles. c) The wavelength shift values of the composite hydrogel films with different bending angles of the finger. d) The relative resistance changes of composite hydrogel film during the bending process of the finger. e) The relative resistance variation of composite hydrogel film under cyclic changes of different angles.

In addition to stretching deformation, such visually dual‐signal composite hydrogel structural color film showed great practical values in wearable strain sensors to monitor human joint motions. Here, we monitored the simulated joint motion by directly attaching the designed film to the puppet's finger. It was demonstrated that the color of the film gradually blue‐shifted from orange to blue‐green along with the increase of bending angle of the finger joints (**Figure** **4**a,b and S9a, Supporting Information). Even during the repeated and continuous movement of the finger, the characteristic reflection peak of the structural color film showed stable shifts (Figure 4c). At the same time, the relative resistance changes during finger bending were also recorded in real‐time. The results revealed that the resistance of the hydrogel film increased or decreased corresponding with the bending angle change of the finger joints (Figure 4d and S9, Supporting Information). Remarkably, the real‐time resistance changes of the film kept stable when the finger was repeatedly bent at the same angle, indicating the stable and sensitive conductivity of composite hydrogel film during dynamic activities (Figure 4e). Therefore, compared with other reported work listed in **Table** **1**, our composite hydrogel structural color film not only had a wide source of raw materials of natural polymer, but also had excellent mechanical properties, conductivity, and unique dual‐signal reporting ability, exhibiting great potential as a bionic electronic skin in practical application.

**Table 1:** A brief overview of recently reported wearable electronic sensors

Materials	Material source	Properties	Monitoring signal	References
Poly(3,4‐ethylenedioxythiophene), and sulfonated graphene oxide	Synthetic polymer	Tough, adhesive, conductive, and biocompatible	Electrical signal	^[^ ^1a^ ^]^
Polyvinyl alcohol, and cellulose nanofibril	Synthetic and natural polymer	Strong and tough, good solvent retention, high ionic conductive, and freezing‐tolerance	Electrical signal	^[^ ^1d^ ^]^
Sodium chloride, sodium alginate, and poly acrylic‐acrylamide	Synthetic and natural polymer	Stretchable, flexible, elastic, stable, and conductive	Electrical signal	^[^ ^1e^ ^]^
Carbon nanotubes, polydopamine, and polyurethane	Synthetic polymer	Stretchable, adhesive, self‐healable, conductive, and dual‐signal monitoring	Electrical and optical signal	^[^ ^4a^ ^]^
Hydroxypropyl cellulose, poly (acrylamide–co‐acrylic acid), and carbon nanotubes	Synthetic and natural polymer	Multiple responsive to pressure, tension, and temperature, conductive, and dual‐signal monitoring	Electrical and optical signal	^[^ ^12a^ ^]^
Silk fibroin, polyacrylamide poly(3,4‐ethylenedioxythiophene):poly (4‐styrene sulfonate), and graphene oxide	Synthetic and natural polymer	Stretchable, conductive, biocompatible, and dual‐signal monitoring	Electrical and optical signal	This work

**Figure 5:**
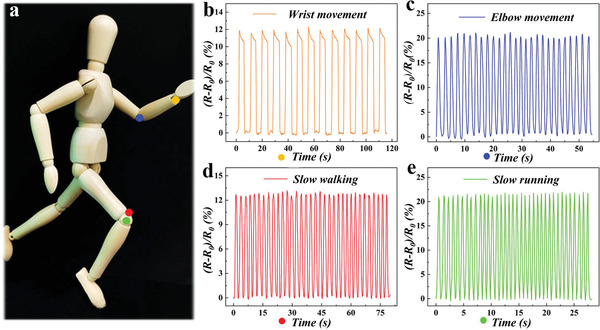
Application of the composite hydrogel film in monitoring the joint motions. a) The photograph of the puppet and the position attached by the film. b–d) Relative resistance variations of the composite hydrogel film with the puppet joint motions by manual operation from its wrist (b), elbow (c), and leg (d,e).

Apart from finger motions, the composite hydrogel structural color film demonstrated its prominent sensitivity and repeatability in practical applications during the movement of other parts of the human body. The composite hydrogel film was placed on the puppet's wrist, elbow, and knee to monitor the activity of each part (**Figure** **5**). It was found that the film exhibited noticeable resistance changes when moving at various parts, and the electrical signal was almost the same in each movement cycle, which proved the sensitivity and stability of the designed film. Notably, the resistance variation happened when the puppet was manually controlled to simulate different running speeds. Simultaneously, according to the deformation caused by the movement of different parts, the characteristic reflection peaks of the film also showed a rapid and stable variation (Figure S10, Supporting Information). These results demonstrated that the human joint motions could be precisely tracked by the film, which possessed stable and sensitive conductivity and self‐reporting ability. And the dual‐signal response performance in terms of real‐time color sensing and electrical signal monitoring suggested that the film could be explored as a potential candidate in monitoring sports.

## 3 Conclusion

In conclusion, we have developed a composite structural color hydrogel film with excellent stretchability, flexibility, conductivity, and superior self‐reporting ability for a dual‐signal response of bionic electronic skin. This system was fabricated through negative replication of colloidal crystal templates by PAM, SF, PEDOT:PSS, and GO mixture. As a result, the composite hydrogel film inherited the unique optical properties and bright structural colors of the colloidal crystal templates while retaining the composite hydrogel's stretchability and flexibility. Besides, superior conductivity was achieved after acid treatment. Notably, structural colors enable the composite hydrogel film to realize real‐time visual monitoring with self‐reporting ability. These features make the designed composite hydrogel structural color film possess great potential in the field of biomimetic electronic skins.

## 4 Experimental Section

### Materials

Silica nanoparticles were self‐synthesized in the laboratory. Silk fibroin (SF) was obtained from Simatech Inc. Acrylamide (AM), *N,N*′‐methylenebisacrylamide (BIS), and 2‐hydroxy‐2‐methyl‐1‐phenyl‐1‐propanone (HMPP) were purchased from Sigma‐Aldrich. The PEDOT:PSS (PP) aqueous suspension (Clevios PH1000) was obtained from Heraeus Electronic Materials GmbH (Leverkusen, Germany). Graphene oxide (GO) was bought from Nanjing XFNANO Materials Tech Co., Ltd. Hydrofluoric acid (HF) and hydroiodic acid (HI) were obtained from Aladdin Industrial Corporation. Water used in all experiments was purified through a Milli‐Q Plus 185 water purification system (Millipore, Bedford, MA) with resistivity higher than 18 MΩ cm.

### Preparation of the Composite Hydrogel with Different Stretchable and Conductive Performance

First, a polyacrylamide (PAM) aqueous solution composed of AM, BIS and HMMP was prepared, in which the concentration of AM was 60 wt %, the mass ratio of AM to BIS was 300:1, and the concentration of HMMP was 1 v/v %. Second, an SF aqueous solution with a concentration of 15% was prepared. Then, SF solution with different volumes was added into the PAM solution and stirred until sufficient mixing, aiming to obtain PAM/SF hybrid hydrogels with the different stretchable abilities for the optimized one. Next, PP and GO solution with different ratios were added into the PAM/PF solution and stirred until complete mixing. Finally, the prepared mixed solution was put into a mold and polymerized under UV light exposure for 1 min.

### Construction of the Composite Structural Color Hydrogel Film

The composite structural color hydrogel film was fabricated using a sacrificial template method. The monodisperse silica nanoparticles synthesized in the laboratory were first washed repeatedly with ultrapure water, and then washed with ethanol 3–5 times to fully replace ultrapure water. Next, an ethanol solution with a concentration of 20% silica nanoparticles was prepared. A simple glass device was used to evenly spread the dispersed silica nanoparticle solution on the glass substrate. During the rapid volatilization of ethanol, silica nanoparticles were vertically deposited and self‐assembled on the glass substrate, and colloidal crystal templates were obtained. Then, to ensure the flatness of the film, another simple device was designed. A certain thickness was created on the blank edges of the nanoparticles‐deposited glass, followed by the covering of another glass, thereby leaving a gap for the entrance of pre‐gel solution. After that, under the action of capillary force, the prepared pre‐gel solution (PAM/SF/PP/GO) was infiltrated into the space between the silica nanoparticles of the colloidal crystal templates, following the polymerization exposed to UV light for 1 min. Finally, the hybrid hydrogel film was immersed in 4% HF to remove the colloidal crystal templates, forming the composite structural color hydrogel film. In addition, to further improve the conductive performance of the composite structural color film, the prepared composite hydrogel film was treated for around one minute by HI solution, and then residual HI was removed by washing with ultrapure water repeatedly.

### Characterizations

Field emission scanning electron microscopy (FESEM, Ultra Plus, Zeiss) was used to characterize the microstructures of colloidal crystal template, hybrid hydrogel, and composite structural color hydrogel film. The stress–strain curves of the samples were obtained by Single Column Motor Meter (500, HSV). Reflection spectra were measured using an optical microscope (Olympus, BX51) equipped with a fiber‐optic spectrometer (Ocean Optics, USB2000‐FLG). Electrical tests were all performed by a SEMICONDUCTOR CHARACTERIZATION SYSTEM 4200‐SCS (KEITHLEY, USA).

## Conflict of Interest

The authors declare no conflict of interest.

## Supporting information

Supporting Information

Supplemental Movie 1

## Data Availability

Research data are not shared.
