# Supplementary material for: Stretchable and Conductive Composite Structural Color Hydrogel Films as Bionic Electronic Skins
Source: Adv Sci (Weinh). 2021 Aug 26;8(20):2102156. doi: 10.1002/advs.202102156 (PMC8529447; doi:10.1002/advs.202102156)
Supplement: Supplementary file 1 — Supporting Information [file ADVS-8-2102156-s002.pdf]

WILEY-VCH

© 2021 Wiley-VCH GmbH

ADVANCED SCIENCE Open Access

Supporting Information

for *Adv. Sci.,* DOI: 10.1002/advs202102156

Stretchable and Conductive Composite Structural Color Hydrogel Films as Bionic Electronic Skins

Hui Zhang, Jiahui Guo, Yu Wang, Lingyu Sun, and Yuanjin Zhao*

Supporting Information

Stretchable and conductive composite structural color hydrogel films as bionic electronic skins

*Hui Zhang, Jiahui Guo, Yu Wang, Lingyu Sun, and Yuanjin Zhao**

Supporting figures

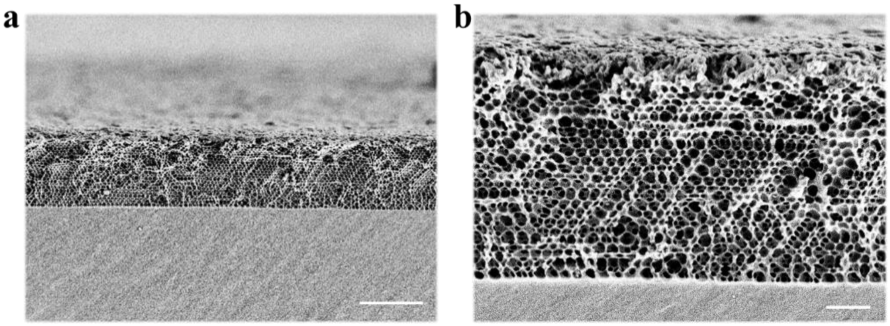

Figure S1. Scanning electron microscope (SEM) image of the cross-section of inverse opal film at different levels of magnification. The scale bars are 4 μm and 1 μm in a and b, respectively.

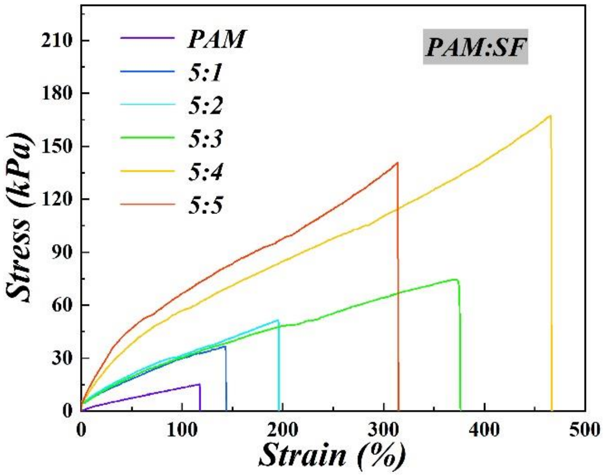

Figure S2. The tensile properties of the PAM/SF composite hydrogels with different volume ratios of PAM and SF.

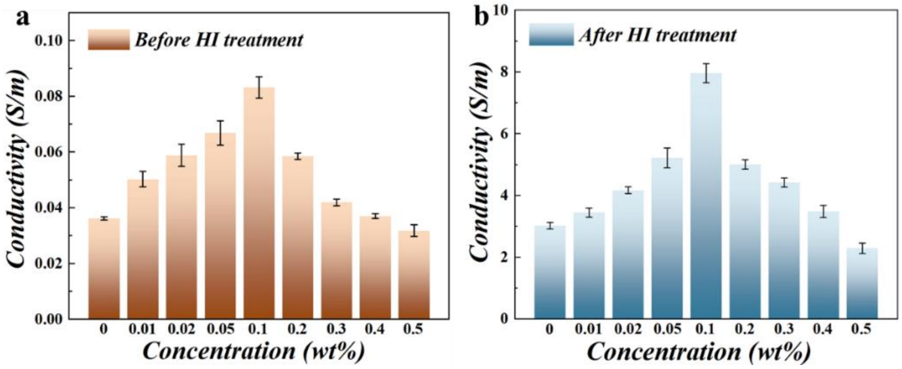

Figure S3. The PAM/SF/PP/GO composite hydrogels' conductivity with different GO contents before (a) and after (b) HI treatment.

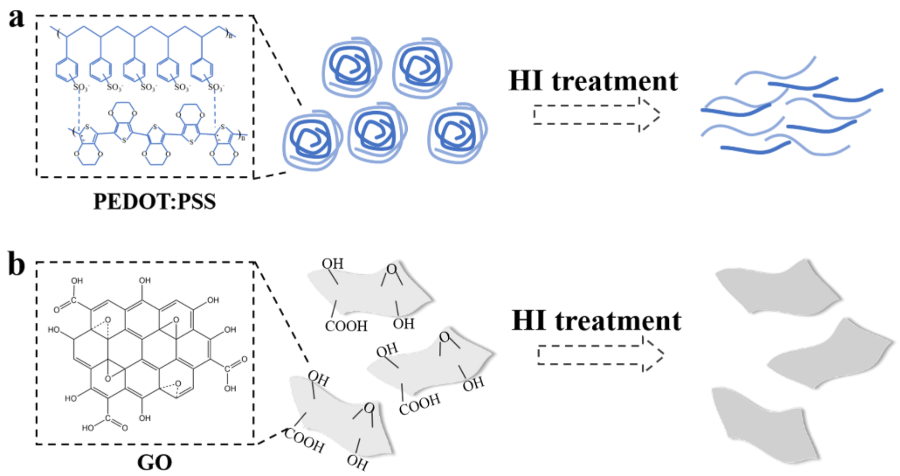

Figure S4. The structural schematic of PEDOT: PSS (a) and GO (b) before and after HI treatment.

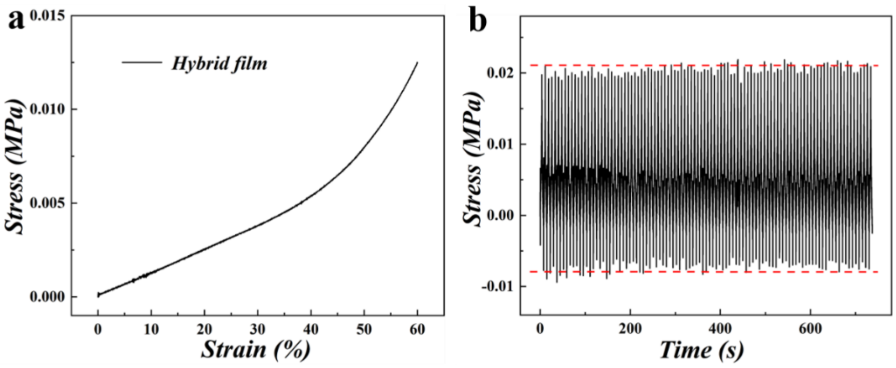

Figure S5. The compressive stress-strain curve (a) and cyclic compressive curve (b) of composite hydrogel film.

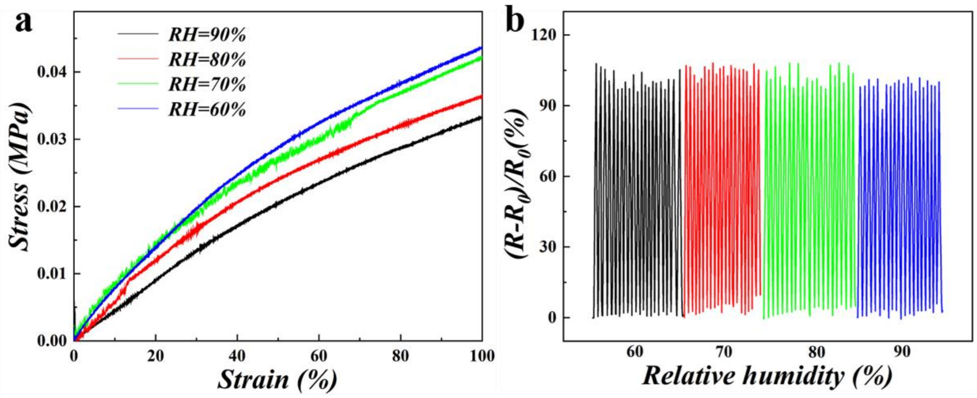

Figure S6. The tensile properties and relative resistance change of composite hydrogel film at different relative humidity (RH).

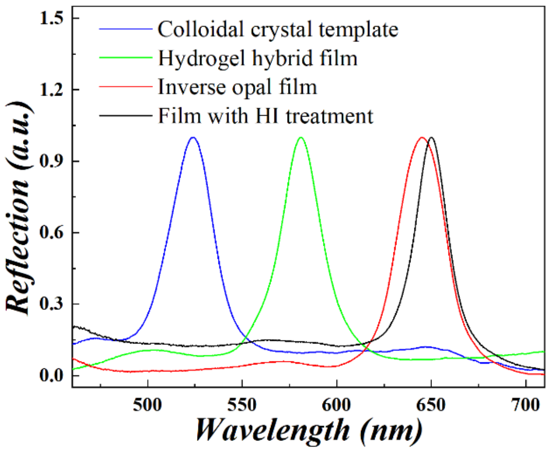

Figure S7. The reflection spectra of colloidal crystal template, hydrogel hybrid film, and inverse opal film before/after HI treatment.

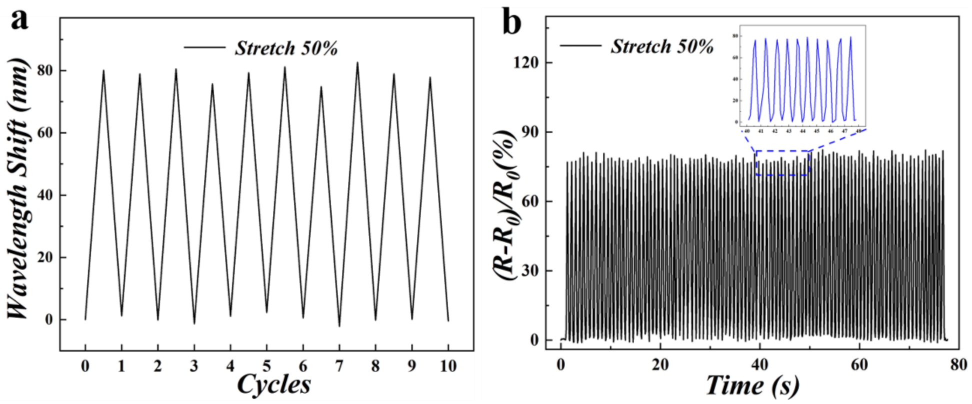

Figure S8. The cycled tests of the wavelength (a) and relative resistance change (b) of composite hydrogel film under 50% stretching.

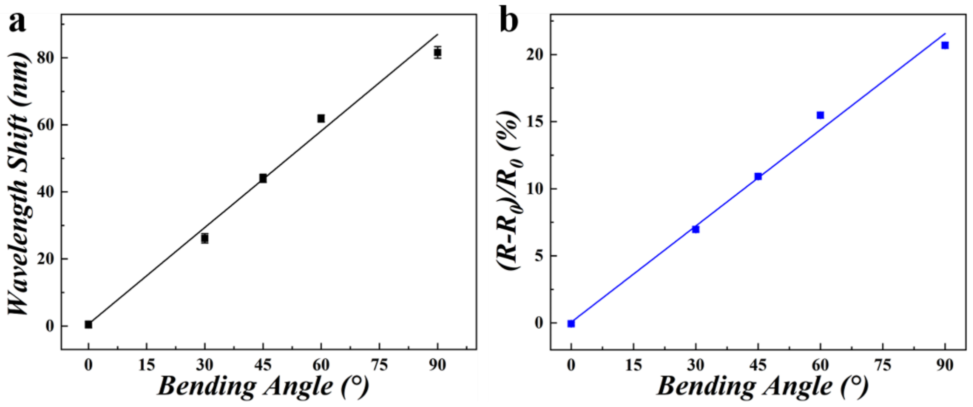

Figure S9. The wavelength shift values (a) and relative resistance change (b) of composite hydrogel film in response to different bending angles.

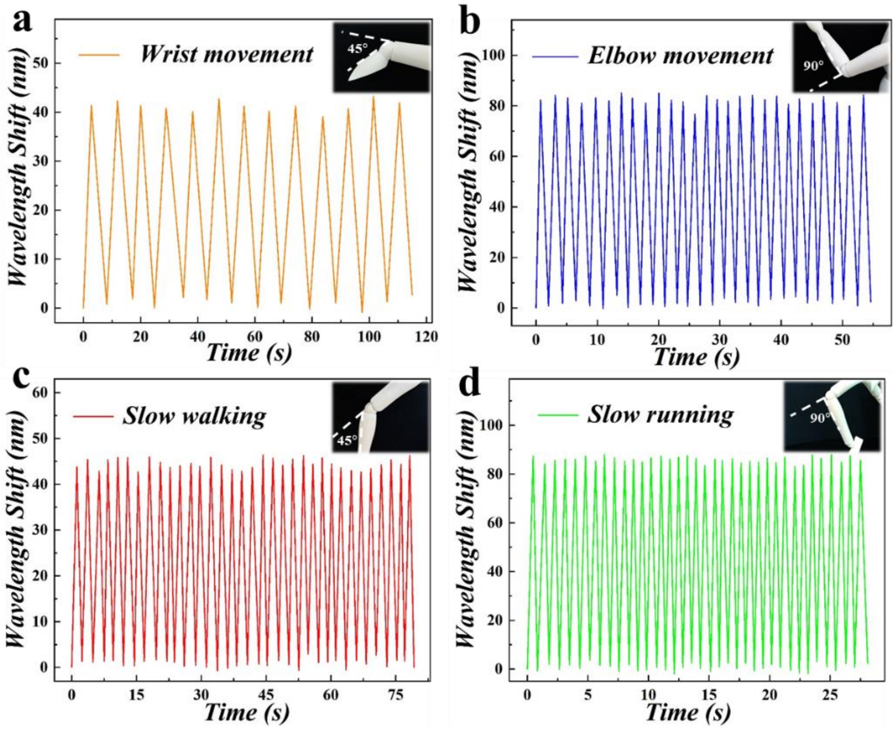

Figure S10. The wavelength shift values of the composite hydrogel film with the puppet joint motions by manual operation from its wrist (a), elbow (b), leg (c, d).

Movie descriptions

Movie S1. 180-degree bending performance of composite hydrogel film.

Author contributions

Y.J.Z. conceived the idea. H. Z. conducted experiments and data analysis. Y. W. and J. H. G. assisted with data analysis and paper writing. L. Y. S contributed to the scientific discussion of the article. H. Z. and Y.J.Z. wrote the manuscript.
